# Codimension-one bifurcation and stability analysis in an immunosuppressive infection model

**DOI:** 10.1186/s40064-016-1737-0

**Published:** 2016-02-01

**Authors:** Zohreh Dadi, Samira Alizade

**Affiliations:** Department of Mathematics, University of Bojnord, Bojnord, Islamic Republic of Iran; Department of Mathematics, Ferdowsi University of Mashhad, Mashhad, Islamic Republic of Iran

**Keywords:** Infectious diseases model, Delay differential equations, Bifurcation theory

## Abstract

One of the important medical problems is infectious diseases such as HIV and hepatitis which annually causes the death of many people. So it is important to study infectious diseases parametric models. In this paper, we investigate differential equations system of HIV and hepatitis (with delay and without delay) from the stability and codimension-one bifurcation point of view. We show that their dynamical behaviour will change when the parameters vary. We prove that this model has a saddle-node bifurcation and transcritical bifurcation when the delay parameter is absent. Also by using the center manifold theory, we show that the delay model has a saddle-node bifurcation.

## Background

Clinical reports have shown that drug treatment in some human pathogens such virus HIV, hepatitis B virus (HBV), and hepatitis C virus (HCV), is not effective. Therefore, designing an optimal drug treatment strategy that leads to sustained immunity has become the essential subject (Shu et al. 2014).

This is the place where mathematical modeling plays an important role as it helps understanding the interactions between viral replication and immune response, (Atangana 2015; Atangana and Alkahtani 2015; Atangana and Goufo 2014; Fenton et al. 2006; Komarova et al. 2003; Li and Shu 2010; Shu et al. 2014).

We consider the mathematical models introduced by Komarova et al. (2003) which the immune response is assumed to be instantaneous in this model. This model is given by two dimensional ordinary differential equations system, as follows

1 $$\left\{ \begin{array}{l} y{'}= y g_r (y) - yz\\ z{'}= z f(y).\\ \end{array}\right.$$

Note that the time lag should not be taken in this model, however, they proved the existence of two stable equilibrium; virus dominant equilibrium (no sustained immunity) and immune control equilibrium (with sustained immunity).

The bistability in this model leads to sustained immunity when the treatment is stopped, because a solution from the basis of the attraction of the virus dominant equilibrium can be lifted to that of the immune control equilibrium via a single phase of therapy.

After that, Shu et al. (2014) incorporated the time lag during the immune response process into Komarova et al.’s model and studied the dynamics between an immunosuppressive infection and antiviral immune response.

To formulate their model, they followed the line in Komarova et al. (2003) and Fenton et al. (2006). They considered the following model

2 $$\begin{aligned} \left\{ \begin{array}{l} y{'}(t) = ry(t) (1-\frac{y(t)}{k}) - ay(t) - p y(t) z(t)\\ z{'}(t) = \frac{c y(t - \tau ) z(t - \tau )}{1+ d y(t - \tau )} - q y(t) z(t) - b z(t), \ \ \\ \end{array}\right. \end{aligned}$$

where *y* and *z* denote the virus population size and population size of immune cells, respectively. The virus population is assumed to grow logistically: *r* is the viral replication rate and *a* is clearance rate. In addition, they assumed virus is killed by immune cells at a rate *pyz* and immune cells are assumed to be inhibited by the virus at a rate *qyz* and died at a rate *b*. The activation rate of immune cells at time *t* is assumed to depend on the virus load and the number of immune cells at time $t- \tau$. Here, $\tau$ is the time lag accounting for the time needed for the immune system to trigger a sequence of events such as antigenic activation, selection and proliferation of immune cells to produce new immune cells. In model 1, it is important to note that *f*(*y*), function of immune expansion by virus load, is considered as follows (Shu et al. 2014)

3 $$f(y) = c \frac{y(t - \tau )}{1+ d y(t-\tau )} - q y(t) - b.$$

Note that if the time lag is ignored, $\tau =0$, model 2 reduces to the following model:

4 $$\begin{aligned} \left\{ \begin{array}{l} y{'}(t) = ry(t) (1-\frac{y(t)}{k}) - ay(t) - p y(t) z(t) \\ z{'}(t) = \frac{c y(t) z(t)}{1+ d y(t)} - q y(t) z(t) - b z(t).\quad \quad \quad \\ \end{array} \right. \end{aligned}$$

They studied the local and global stability of the most of equilibria. By using bifurcation theory, they only found Hopf bifurcation in the model when $\tau = \tau _{bif}$.

In this paper, we follow the line in Shu et al. (2014). It should be noted that, we detect another equilibrium point which is not considered in Shu et al. (2014). Furthermore, we choose another parameters, *r* and *c*, as bifurcation parameters. The parameter *r* is the viral replication rate and the parameter *c* is a coefficient in the function of immune expansion by virus load. We consider *r* and *c* as bifurcation parameters and obtain the following result:

1. (*i*) if $r=r_{bif}$, then the transcritical bifurcation occurs in system 4,
2. (*ii*) if $c = c_{bif}$, then the saddle-node bifurcation occurs in system 4,
3. (*iii*) if $c = c_{bif}$, then the saddle-node bifurcation occurs in system 2.

As we mentioned, Shu et al. (2014) only investigated Hopf bifurcation by considering $\tau$ as bifurcation parameter. But we find new equilibrium in their model and obtain new dynamical behaviours in the model. Furthermore, we find other important parameters in studying dynamics of this model. To the best of our knowledge, this is the first time that these results are obtained in this immunosuppressive infection model.

The rest of paper is organized as follows. In the next section, we obtain the necessary condition of existence of equilibria in immunosuppressive infection model. In “Dynamics of the model without delay (system 4)” section, we will consider the dynamics of model 4. The dynamical behaviour of model 2 is investigated in “Dynamics of the model with delay (system 2)” section. In “Numerical simulation” section, the validity of the main results is illustrated by numerical simulations. Finally, we state some main conclusions.

## Existence of equilibrium points

For any $\tau > 0$, let $C:= \lbrace \phi : [ - \tau , 0 ] \rightarrow R \; is \; continuous\rbrace$ be Banach space of continuous function on $[- \tau , 0]$ with the norm is defined as $\Vert \phi \Vert = \sup _{- \tau \le \theta \le 0} \phi ( \theta )$. We denote the nonnegative cone of *C* by $C^{+}$.

Shu et al. (2014) showed that system 2 with any initial condition $(\phi , \psi ) \in C^{+} \times C^{+}$ admits an unique solution and the solution (*y*(*t*), *z*(*t*)) remains nonnegative for $t \ge 0$ and is bounded in $C^{+} \times C^{+}$. Furthermore, they showed that the bounded region

5 $$\Gamma =\left\{ (\phi , \psi ) \in C^{+} \times C^{+} : \Vert \phi \Vert \le K, \phi (- \tau ) + \frac{p}{c} \psi (0) \le \frac{r K}{\mu } \right\} ,$$

where $\mu = \min \lbrace a, b \rbrace > 0$, is positively invariant with respect to system 2 and the system is well posed (Shu et al. 2014).

Now we find the equilibria of system 2. We then investigate their stability. As we said in “Background” section, we obtain an equilibrium point that it is not considered in Shu et al. (2014).

Cleary $E_0 =(0,0)$ is a trivial equilibrium of system 2, this equilibrium means that any virus cell and immune response do not exist in the body. There exists an equilibrium $E_1 = (\bar{y} , 0) = (\frac{k(r-a)}{r} , 0 )$ provided $r> a$. At equilibrium $E_1$ does not exist any immune response, also viruses are with positive size. Therefore, we call the equilibrium $E_1$ the virus dominante equilibrium (VDE). Assume that $E^{*} = (y^{*} , z^{*})$ is another equilibrium point of system 2 with $y ^{*} >0$ and $z^{*} >0$ which means immune response and virus cells are present at the same time. Therefore, the virus cells can be controlled. Now, we consider the following equations

6 $$\begin{aligned} \left\{ \begin{array}{l} r(1- \frac{y^{*}}{k}) - a - p z^{*}=0 \\ \frac{c y^{*}}{1 + d y^{*}} - q y^{*} - b =0.\\ \end{array}\right. \end{aligned}$$

The first equation of 6 follows that

7 $$z^{*} = \frac{r(k-y^{*}) - ak}{pk} > 0,$$

or other words

8 $$r (k - y^{*}) - ak > 0,$$

then $y^{*} < \bar{y}$ . From the second equation of 6, we have the following function

9 $$g(y) = q d y^{2} - (c- q - bd) y + b.$$

**Fig. 1:**
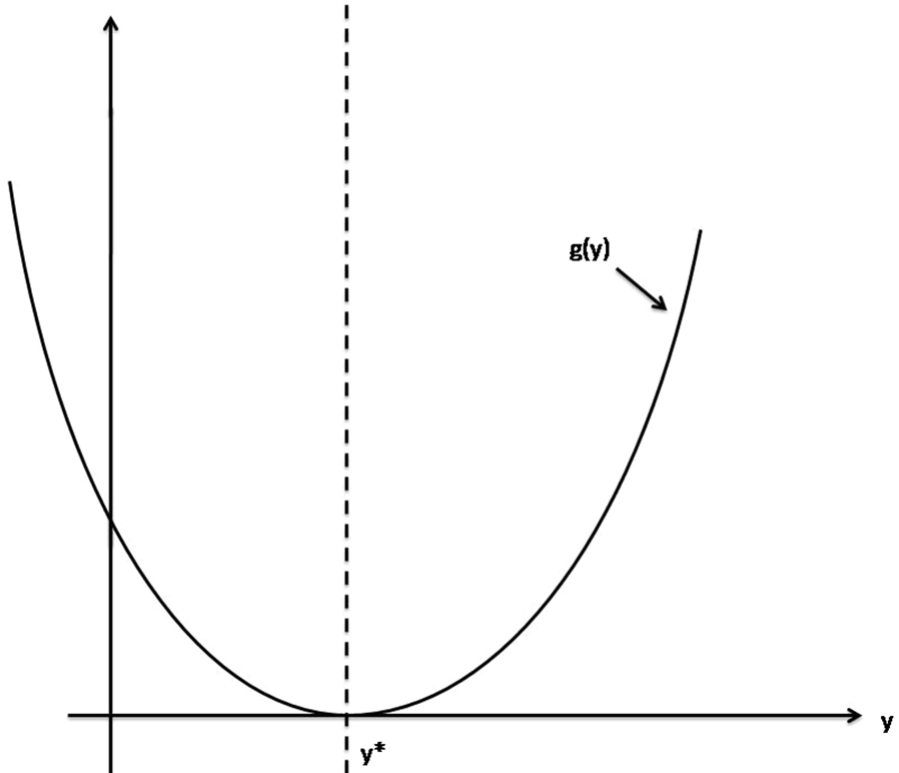
The curves of $g(y) = qdy^{2} - (c-q-bd) y +b$

It is clear that $E^{*}$ exists if and only if $y^{*}$ is the positive root of *g* (*y*) where $y^{*} < \bar{y}$ (Fig. 1).

Shu et al. (2014) investigated the existence of positive roots of *g*(*y*) when $c> (\sqrt{q} + \sqrt{bd})^{2}$. We obtain new results on positive roots of *g*(*y*) when $c= (\sqrt{q} + \sqrt{bd})^{2}$.

### *Remark 1*

$H_1$: if $c= (\sqrt{q} + \sqrt{bd}) ^{2}$, then *g* (*y*) has a double positive root that it is same vertex of parabola,

10 $$y^{*}=\frac{c-q-bd}{2qd}.$$

Now, by defining the threshold values as follows

11 $$\begin{aligned} r_{t}= \left\{ \begin{array}{ll} \frac{ak}{k- y^{*}} & y^{*}< k \\ \infty & y^{*}\ge k, \\ \end{array} \right. \end{aligned}$$

we have the following Lemma.

### **Lemma 1**

*By considering*$H_1$, *the following cases occur*

**Fig. 2:**
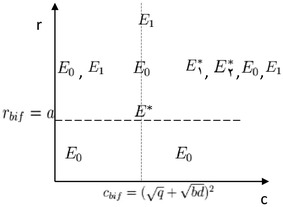
Existence of equilibria of system 2 and 4 in parametric space (*c*, *r*)

- *if*
  12 $$r \le a$$
  *holds, then the equilibrium*$E_0 = (0,0)$*is the only equilibrium*,
- *if*
  13 $$a<r \le r_{t} \; (i.e \; a<r\; \& \; y^{*} \ge \bar{y})$$
  *holds, then there are two equilibria:*$E_0$ and $E_1 =( \bar{y}, 0)$, *where*$\bar{y}=\frac{k(r-a)}{r}$,
- *if*
  14 $$r > r_{t} \; (i.e \; a<r \; \& \; y^{*} < \bar{y})$$
  *holds, then there are three equilibrium*: $E_0$, $E_1$, $E^{*} = (z^{*}, y^{*})$ where $z^{*} =\frac{r(k-y^{*}) - ak}{pk}$ (Fig. 2).

## Dynamics of the model without delay (system 4)

In this section, we provide a complete description about dynamics of system 4. To this end, we begin with the following result on local stability of system 4.

### **Lemma 2**

*Assume that*$H_1$*is satisfied*.

- *If*12*holds, then equilibrium*$E_0$*is locally stable*.
- *If*13*holds, then*$E_0$*is unstable (saddle point) and*$E_1$*is locally asymptotically stable*.
- *If*14*holds, then*$E_0$*is unstable (saddle point)*, $E_1$*is locally asymptotically stable and*$E^{*}$*is locally stable*.

### *Proof*

Suppose that $(\tilde{y}, \tilde{z})$ is an equilibrium of system 4. The associated characteristic equation is given by

15 $$g_0 (\xi ) = \xi ^{2} + c_1 \xi + c_0$$

where

16 $$c_1 = - \left( r - \frac{2r}{k} \tilde{y} - a - p \tilde{z} + \frac{c \tilde{y}}{1+ d \tilde{y}} - q \tilde{y} - b\right)$$

and

17 $$c_0 = \left( r- \frac{2r}{k} \tilde{y} -a -p \tilde{z}\right) \left( \frac{c \tilde{y}}{1+ d \tilde{y}} - q \tilde{y} - b\right) +p \tilde{y} \left( \frac{c \tilde{z}}{(1+d \tilde{y})^{2}} - q \tilde{z}\right) .$$

Then characteristic equation 15 at $E_0$ has two roots, $\xi _1 =-b <0$ and $\xi _2 =- (a-r)$ . If 12 holds, then $E_0$ is stable and if 13 or 14 holds then $E_0$ is saddle point.

Also, characteristic equation 15 at $E_1 = (\bar{y} , 0)$ has two roots,

18 $$\xi _1 = -r+a, \quad \xi _2 =- \frac{g (\bar{y})}{1+d \bar{y}}.$$

Note to the graph of *g*(*y*), it is obvious that $g(\bar{y}) >0$. Now, if 13 or 14 holds, then the equilibrium $E_1$ is asymptotically stable. We suppose that 15 holds, by substituting $E^{*}$ at Eq. 15, we have

19 $$c_0 = p y^{*} z^{*} \left(- q + \frac{c}{(1+ d y^{*})^{2}}\right), \quad c_1 = \frac{r}{k} y^{*} > 0.$$

Suppose that

20 $$g _1 (y) = - q + \frac{c}{{(1 + d y)}^{2}}.$$

It is clear $g_1(\hat{y})=0$, where $\hat{y} = \frac{\sqrt{c} - \sqrt{q}}{d \sqrt{q}}$. Condition $H_1$ follows that $\hat{y} = y^{*}$, then $g_1 (y^{*}) = 0$. Therefore, the roots of Eq. 15 are $\xi _1 = 0$, $\xi _2 = - \frac{r}{k} y^{*}$, or other words $E^{*}$ is locally stable.

When $r \ge a$, the infection can not spread in body of patient, so there is no virus cell and immune response. In this case, system 4 converges to $E_0$. We know viral cells infect the host without immune response as *r* increases from *a* to $r_t$. In this case, system 4 converges to $E_1$ and the equilibrium point $E_1$ is locally asymptotically stable. By increasing *r* from $r_t$, immune response increases and controls viral cells. In this case, $E^{*}$ and $E_1$ exist. Therefore, to obtain the better conditions and control of virus cells, we should converge the system to the equilibrium point $E^{*}$.

### **Lemma 3**

*Assume that*$H_1$*is satisfied, therefore system*4*has a saddle node bifurcation at equilibrium*$E^{*}$*when the parametercvaries*.

### *Proof*

By Lemma 2, characteristic equation 15 at $E^{*}$ has two simple roots $\xi _1 =0$ and $\xi _2 =-\frac{r}{k} y^{*}$. Therefore $(E^{*} , c)$ is a bifurcation point where $c= c_{bif} =(\sqrt{q} + \sqrt{bd})^{2}$. Assume that $A= Df (E^{*}, c_{bif})$, then the eigenvectors of *A* and $A^{T}$ at zero eigenvalue are

21 $$\begin{aligned} V= \left[ \begin{array}{l} -\frac{pk}{r}\\ 1 \end{array}\right] , \quad W= \left[ \begin{array}{l} 0\\ 1 \end{array}\right] , \end{aligned}$$

hence we have

22 $$\begin{aligned}&(a){:} \quad W^{T} f_c (E^{*}, c_{bif}) = \frac{y^{*}z^{*}}{1+dy^{*}}, \end{aligned}$$

23 $$\begin{aligned}&(b){:} \quad W^{T} [ D^{2}f(E^{*}, c_{bif}) (V,V)] = - \frac{2cdp^{2} k^{2} z^{*}}{r^{2} (1+d y^{*})^{3}} \end{aligned}$$

where the two conditions (a) and (b) are opposed zero. By Sotomayor Theorem (Guckenhiemer and Holmes 1993; Perko 1991), system 4 has a saddle-node bifurcation at $E^{*}$ whene $c= c_{bif}$.

### **Lemma 4**

*If*$H_1$*is satisfied, then system*4*has a trancscritical bifurcation at equilibrium*$E_0$*when*$r=r_{bif}=a$.

### *Proof*

By Lemma 2, characteristic equation 15 has two roots $\xi _1 = - b$ and $\xi _2 = - (a-r)$. Therefore $(E_0, r_{bif})$ is a bifurcation point where $r_{bif} = a$ and $\xi _2 =0$ is a simple zero of 15. Now, we assume $A= Df(E_0, r_{bif})$ then the eigenvectors of *A* and $A^{T}$ at zero eigenvalue are

24 $$\begin{aligned} V= \left[ \begin{array}{l} 1 \\ 0 \end{array}\right] , \quad W= \left[ \begin{array}{l} -1 \\ 0 \end{array}\right] . \end{aligned}$$

Therefore, we have the following quantities

25 $$\begin{aligned}&(a){:}\quad W^{T} f_r (E_0, r_{bif}) = 0, \end{aligned}$$

26 $$\begin{aligned}&(b){:}\quad W^{T} [ Df_r (E_), r_{bif})V] = -1\ne 0, \end{aligned}$$

27 $$\begin{aligned}&(c){:}\quad W^{T} [ D^{2}f(E_0, r_{bif}) (V,V)] = - \frac{2a}{k}\ne 0. \end{aligned}$$

By Sotomayor Theorem (Guckenhiemer and Holmes 1993; Perko 1991), system 4 has a trancscritical bifurcation at $E_0$ when $r_{bif} = a$.

According to Lemma 4, we know that system 4 has a transcritical bifurcation at $E_0$, when $r= r_{bif}$. For $r \le r_{bif}$, only equilibrium point $E_0$ is stable. In this case, the patient$^{,}$s body does not have virus cells and immune response. Also, with increasing *r* ($r > r_{bif}$), the equilibrium $E_1$ occurs; in this case the system has a branch of stable equilibrium $E_1$ and a branch of the unstable equilibrum $E_0$ that express the transcritical bifurcation. In the branch of the stable equilibirum $E_1$, the patient has a viral cells without any immune response. Therefore as shown if the viral replication rate *r* is greater than the threshold $r_t$, then the two equilibrium points $E_1$ and $E^{*}$ at the same time are stable and the bistability phenomenon occurs. Also, we know that for $c < c_{bif}$, there is no equilibrium $E^{*}$ and according to assumption $H_1$ at $c= c_{bif}$, the equilibrium $E^{*}$ will be found. After passing through $c_{bif}$ ($c > c_{bif}$); according to Shu et al. (2014), the system has two equilibrium $E_1 ^{*}$ and $E_2 ^{*}$. This means that there is a saddle-node bifurcation. With finding quantity of bifurcation parameter and rising it, we should try the patient’s condition set in the stable branch of saddle-node bifurcation. In this case virus cells are controlled and patient is in the path of recuperation.

## Dynamics of the model with delay (system 2)

In this section, we would like to investigate dynamics of system 2 with $\tau > 0$.

### Stability of equilibria

The first, we study the equilibrium $E_0$ in following theorem.

#### **Theorem 1**

*if*$r\le a$, *then*$E_0$*is locally stable; while if*$r > a$*then*$E_0$*is unstable*.

#### *Proof*

By computing the characteristic equation of system 2 at $E_0$, we have

28 $$(\xi - (r -a)) (\xi +b) = 0.$$

It complets the proof.

Now, we consider the characteristic equation associated with the linearization of system 2 at $E_1$

29 $$(\xi + r - a) \left( \xi +q \bar{y} + b - \frac{c\bar{y}}{1+ d \bar{y}} e^{- \xi \tau }\right) = 0.$$

Note that $E_1$ exists only if $r>a$, thus one root is $\xi _1=a-r<0$. Therefore, the dynamic of $E_1$ is depend on distribution of roots of the following equation

30 $$g_2 (\xi ) = \xi + q \bar{y} + b - \frac{c \bar{y}}{1+ d \bar{y}} e^{- \xi \tau }.$$

#### **Theorem 2**

*The equilibrium point*$E_1$*is locally asymptotically stable*.

#### *Proof*

By Lemma 2, the conclusion is true for $\tau =0$. We have to prove that all roots of $g_2 (\xi )$ have only negative real parts. Suppose that $\xi = \alpha + i \omega$ is a zero of $g_2 (\xi )$. After substituting in $g_2(\xi )$, we obtain

31 $$\begin{aligned}&\left\{ \alpha + b + q \bar{y} - \frac{c \bar{y}}{1+d \bar{y}} (\cos \omega \tau ) e^{- \alpha \tau } \right\} +i \left\{ \omega + \frac{c \bar{y}}{1+d \bar{y}} (\sin \omega \tau ) e^{- \alpha \tau } \right\} =0. \end{aligned}$$

Therefore

32 $$(\alpha + b + q \bar{y}) ^{2} + \omega ^{2} = e^{-2\alpha \tau } \left( \frac{c \bar{y}}{1+d \bar{y}}\right) ^{2},$$

Note that $\alpha \ne 0$, by the above discussion, we assume $\alpha > 0$. The right hand side convergent to zero but left hand side is perfectly elder of zero. Therefore, we have a contradiction or $\alpha < 0$ and the proof is complete.

Theorems 1 and 2 and Lemma 1 show that if 14 holds, then $E_0$ is unstable and $E_1$ is stable, and $E^{*}$ exists. We now study the stability of $E^{*}$. The characteristic equation at $E^{*}$ is

33 $$G(\xi ) = \xi ^{2} + a_1 \xi + a_0 + (b_1 \xi + b_0) e^{- \xi \tau } = 0$$

where

$$\begin{aligned} a_1 &= {} g y^{*} + b + \frac{r y^{*}}{k}, \\ a_0 &= {} (q y^{*} + b) \frac{r y^{*}}{k}- pqy^{*} z^{*},\\ b_1 &= {} -(q y^{*} + b), \\ b_0 &= {} - (q y^{*} + b) \frac{r y^{*}}{k} + \frac{pcy^{*} z^{*}}{(1+ d y^{*})^{2}}. \end{aligned}$$

By Lemma 2, when $\tau = 0$, $E^{*}$ is asymptotically stable, i.e, all roots of the characteristic equation 33 have negative real parts. We want to prove $E^{*}$ is locally stable. With inverse process, we suppose that $i \omega \; (\omega > 0)$ is the root of $G (\xi )$, then we have

$$\begin{aligned} \omega ^{2} - a_0 & = {} b_1 \cos \omega \tau +b_1 \omega \sin \omega \tau \\ - a_1 \omega & = {} b_1 \omega \cos \omega \tau - b_0 \sin \omega \tau , \end{aligned}$$

which yields

34 $$F (\omega ) = \omega ^{4} + (a_1 ^{2} - 2a_0 - b_1 ^{2}) \omega ^{2} + (a_0 ^{2} - b_0 ^{2}) =0,$$

where

$$\begin{aligned} c_1^{\prime}& := {} a_1^{2} - 2a_0 - b_1^{2}= \left( \frac{ry^{*}}{k}\right) ^{2} + 2pqy^{*}z^{*} > 0, \\ c_0^{\prime} & := {} a_0^{2} - b_0^{2} \\& = {} py^{*2} z^{*} g_1 (y^{*})\left( -pqz^{*}- \frac{pcz^{*}}{1+dy^{*}}+ \frac{2r(qy^{*}+b)}{k}\right) . \end{aligned}$$

Since $g_1 (\hat{y}) = g_1(y^{*}) =0$, thus $c_0^{\prime} =0$, and

35 $$F(\omega ) = (\omega ^{2}) ^{2} + c_1^{\prime} \omega ^{2} =0.$$

Therefore, Eq. 33 has non purely imaginary root. On the other hand, we know $g_1 (y^{*}) = 0$ or $\frac{c}{(1+d y^{*})^{2}} = q$. Therefore $a_0 + b_0 = 0$, and $\xi = 0$ is a simple zero of $G(\xi )$. Now we can state the following theorem.

#### **Theorem 3**

*Roots of characteristic equation*33*have negative real parts other than*$\xi =0$, *if*

1. $$a_0 >0$$
2. $$a_1^{2} - 2a_0 >0$$.

*Hence*,  $E^{*}$*is locally stable*.

#### *Proof*

Suppose that $\xi = \alpha + i \omega$ is a zero of 33. After substituting it in 33, we obtain

$$\begin{aligned} \lbrace \alpha ^{2} - \omega ^{2}+ & {} a_1 \alpha + a_0 + e ^{- \alpha \tau }((b_1 \alpha + b_0) \cos \omega \tau + b_1 \omega \sin \omega \tau )\rbrace + \\ i \lbrace z \alpha \omega+ & {} a_1 \omega + e ^{- \alpha \tau } (b_1 \omega \cos \omega \tau - (b_1 \alpha + b_0) \sin \omega \tau ) \rbrace =0. \end{aligned}$$

Therefore

$$\begin{aligned}&(\alpha ^{2} + \omega ^{2}) + (a_1 \alpha + a_0)^{2} + 2 (a_1 \alpha + a_0) (\alpha ^{2} + \omega ^{2}) + \omega ^{2}(a_1 ^{2} - 4 a_0)\\&\quad = e^{-2 \alpha \tau } [ b_1 ^{2} (\alpha ^{2} + \omega ^{2}) + b_0 (b_0 + 2 b_1 \alpha ) ]. \end{aligned}$$

Note that $\alpha \ne 0$. By the above discussion, we assume $\alpha > 0$. By conditions of (1) and (2) , the right hand side is convergent to zero but left hand side is perfectly elder of zero. Hence, we have a contradiction or $\alpha < 0$. This completes the proof.

### Saddle-node bifurcation of system 2

In this subsection, we want to study codimension-one bifurcations of system 2. For this aim, we consider *c* as bifurcation parameter. By Remark 1, we know that $E^{*}$ exists if $c= (\sqrt{q} + \sqrt{bd})^{2}$. Also, we know that $E^{*}$ is locally stable by Theorem 3, and codimension-one bifurcation can occur in system 2 at $E^{*}$. Define $c_{bif} = (\sqrt{q} + \sqrt{bd})^{2}$. Now, we assume $\mu = c - c_{bif}$ as bifurcation parameter and rewrite system 2 as follows

36 $$\begin{aligned} \left\{ \begin{array}{l} y{'}(t) = ry(t) (1-\frac{y(t)}{k}) - ay(t) - p y(t) z(t), \\ z{'}(t) = \frac{(\mu + c_{bif}) y(t - \tau ) z(t - \tau )}{1+ d y(t - \tau )} - q y(t) z(t) - b z(t),\\ \mu {'} = 0. \end{array} \right. \end{aligned}$$

Below we state the important theorem about existence saddle-node bifurcation of system 36. For this aim, we use center manifold theory of DDE, see “Appendix”.

#### **Theorem 4**

*System*36*has a saddle-node bifurcation at*$E_{new}^{*} = (y^{*} , z^{*} , 0)$*and*$\mu =0$, *if*$qpkdy^{*} (r+(qy^{*}+b - pqkz^{*})\tau )\ne 0$.

#### *Proof*

We consider the linearization of system 36 at $E_{new}^{*}$

37 $$\begin{aligned} \left[ \begin{array}{l} \dot{y}(t)\\ \dot{z}(t)\\ \dot{\mu }(t) \end{array}\right] = A_0 \left[ \begin{array}{l} y(t)\\ z(t)\\ \mu (t) \end{array}\right] + A_1 \left[ \begin{array}{l} y(t - \tau )\\ z(t - \tau )\\ \mu (t - \tau ) \end{array}\right] \end{aligned}$$

where

38 $$\begin{aligned} A_0 = \left[ \begin{array}{lll} r - \frac{2r}{k} y^{*} - a - p z^{*} &{} -p y^{*} &{} 0\\ -q z^{*} &{} -q y^{*} - b &{} \frac{y^{*} z^{*}}{1+ d y^{*}}\\ 0 &{} 0 &{} 0 \end{array}\right] , \quad A_1 = \left[ \begin{array}{lll} 0 &{} 0 &{} 0\\ \frac{ c_b z^{*}}{(1+ d y^{*})^{2}} &{} \frac{ c_b y^{*}}{(1+ d y^{*})} &{} 0 \\ 0 &{} 0 &{} 0 \end{array}\right] . \end{aligned}$$

The characteristic equation associated with system 37 is

39 $$G_2 (\xi )= \xi . G(\xi ) = \xi ^{3} + a_1 \xi ^{2} +a_0 \xi +(b_1 \xi ^{2} + b_0 \xi ) e^{- \xi \tau } = 0$$

where $G(\xi )$ is defined by 33.

By Theorem 3, $G(\xi )$ has $\xi =0$ as a root. Thus, $G_2 (\xi )$ has double zero roots. We want to obtain the center manifold associated with 37. To this end, we compute basis of a center subspace associated with 37 and adjoint system as follows

40 $$\begin{aligned} \phi = \left[ \begin{array}{ll} \frac{-pk}{r} &\quad{} \frac{p k^{2} z^{*}}{r^{2} (1+ dy^{*})}\\ 1 &\quad{} 0 \\ 0 &\quad{} 1 \end{array}\right] , \quad \psi {'}= \left[ \begin{array}{lll} 0 &\quad{} 0 &\quad{} 1\\ 0 &\quad{} 1 &\quad{} 0 \end{array}\right] . \end{aligned}$$

By using inner multiplication, we have

41 $$\begin{aligned} \langle \psi {'} , \phi \rangle = \left[ \begin{array}{ll} 0 &{} 1 \\ 1+(qy^{*}+b -\frac{pqk z^{*}}{r})\tau &{} \frac{pqk^{2} z^{{* 2}}}{r^{2}(1+dy^{*})} \tau \\ \end{array}\right] . \end{aligned}$$

With normalization $\psi$ relation to $\phi$, we obtain

42 $$\begin{aligned}\psi = \left[ \begin{array}{lll} 0 &\quad{} \bar{A}_1 &\quad{} \bar{A}_2\\ 0 &\quad{} 0 &\quad{} 1 \end{array}\right] , \end{aligned}$$

where

43 $$\begin{aligned}\bar{A}_1 & = {} \frac{r}{r+(qy^{*}+b - pqkz^{*}) \tau } , \end{aligned}$$

44 $$\begin{aligned}\bar{A}_2 & = {} \frac{pqk^{2} z^{* 2} \tau }{r(1+dy^{*})(r-pqkz^{*}\tau )+r^{2} cy^{*}\tau } \end{aligned}$$

then

45 $$\langle \psi , \phi \rangle = I_{2}.$$

Now, suppose that local coordinates at center manifold is $U = (u, \mu ) ^{T}$. The terms of nonlinear system 36 and matrix *B* are

46 $$B = \left[ \begin{array}{ll} 0 &\quad{} 0\\ 0 &\quad{} 0 \end{array}\right] ,$$

$$\begin{aligned} F (y, z , \mu ) & = {} \left(\frac{2r}{k} y^{*} y(t)- \frac{r y^{2}(t)}{k} + p (z^{*} y(t) + y^{*} z(t)) - p y(t) z(t) + y^{*}(a-r) \right.\\&\quad , \frac{(c_b + \mu ) y(t - \tau ) z(t - \tau )}{1+ d y(t- \tau )} - q y(t) z(t) + q (y^{*} z(t) + z^{*} y(t))\\&\quad \left.- \frac{y^{*} z^{*} \mu (t) }{1+d y^{*}} - \frac{c_b z^{*} y(t - \tau )}{(1+ d y^{*})^{2}} - \frac{c_b y^{*} z(t- \tau )}{1+ d y^{*}},0 \right) ^{T} \end{aligned}$$

Therefore, we have the following system by using the center manifold

47 $$\begin{aligned} \left\{ \begin{array}{l} u{'}=\frac{1}{\bar{A}_1} [\left( -\frac{2qpkdy^{*}}{r}\right) u^{2} + \left( \frac{2qpdk^{2} y^{*}z^{*}}{r^{2} (1+d y^{*})}\right) u \mu - \left( \frac{y^{*}z^{*}}{1+dy^{*}}\right) \mu - b u] \\ \\ \mu {'} = 0.\\ \end{array} \right. \end{aligned}$$

Define

48 $$\begin{aligned} A:=- \frac{2qpkdy^{*}}{r \bar{A}_1}, \quad B:=\frac{2qpdk^{2} y^{*}z^{*}}{r^{2} (1+d y^{*}) \bar{A}_1}, \quad C:= -\frac{y^{*}z^{*}}{ \bar{A}_1 (1+dy^{*})}, \quad D:=\frac{-b}{\bar{A}_1}, \end{aligned}$$

and

49 $$\mu _1 :=\mu , \qquad \mu _2 :=B \mu + D.$$

Then

50 $$u{'}= A u^{2} + \mu _2 u + C \mu _1.$$

Also, by assumption $u_{new}= u - \frac{\mu _2}{A^{2}}$, we have

51 $$u{'}_{new}= Au_{new}^{2} + \mu _{new}$$

where

52 $$\mu _{new} = C\mu _1 - \frac{\mu _2 ^{2}}{4A}$$

Thus, studying dynamics of system 36 is equivalent to studying the following system

53 $$\begin{aligned} \left\{ \begin{array}{l} u{'}_{new}= Au_{new}^{2} + \mu _{new} \\ \mu {'}_{new} = 0. \\ \end{array}\right. \end{aligned}$$

Hence, by assumption of theorem, system 53 or other words system 36 has a saddle-node bifurcation.

## Numerical simulation

**Fig. 3:**
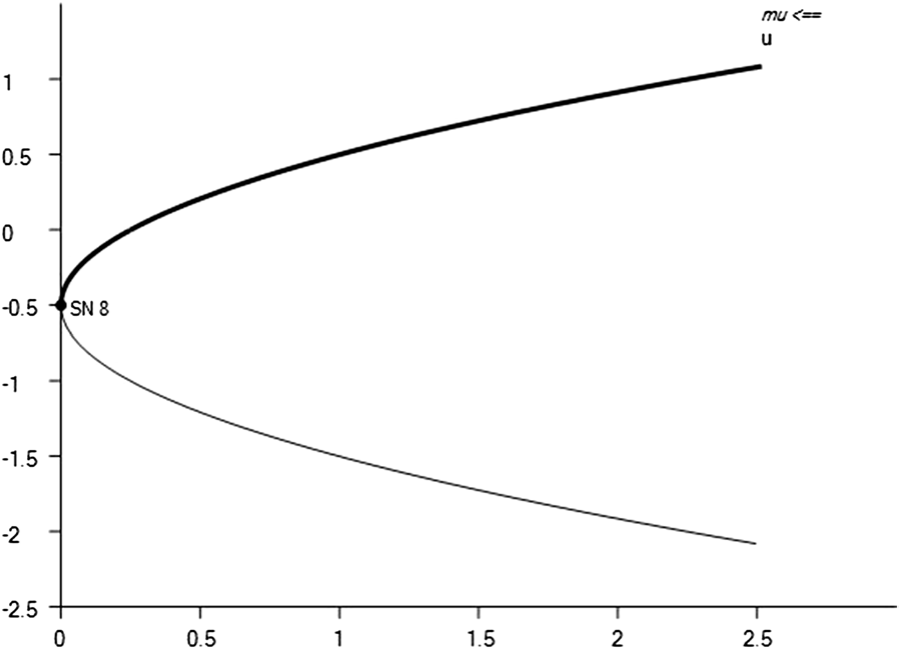
Saddle-node bifurcation diagram

By considering the following parameters:

54 $$\begin{aligned} y^* =1.003, \ z^* = 1.003, \ A=1.012,\ B=0.25\ D=-1, \ \mu = 0.249,\ \mu _2= -1 \end{aligned}$$

we have the saddle-node bifurcation in system 36, see Fig. 3.

## Conclusion

An immunosuppressive infection model with discrete delays and without delay is considered. We have analyzed this model without delay in this paper and showed that the model has transcritical and saddle-node bifurcation at different parameters. We obtained a new equilibrium in our model with delay. Then, we have shown that this model undergoes saddle node bifurcation at this equilibrium. We then compute its normal form. Finally, the presented numerical simulations have demonstrated the correctness of the theoretical analysis.

## Appendix (Center manifold for DDES)

We consider the center manifold theory is stated in Dadi et al. (2012). In this section, we briefly state the center manifold theory for DDEs with parameters. For more details, one can refer to Balachandran and Kalmar-Nagy (2009), Hale and Lunel (1993) and Hale (1977). Consider the general delay-differential equation

55 $$\dot{Y}(t)=g(X(t),X(t-\tau ), \mu )$$

where $Y=(X,\mu ),\ \ \mu \in R, \ X\in R^2$ and $\tau >0$. We shall assume that *g* is $C^r$, for *r* large enough and the equation admits zero as the equilibrium. Note that the Eq. (55) should be viewed as the suspended system where the parameter $\mu$ is included as trivial dynamic ($\dot{\mu }=0$). We separate the system (55) to the linear and nonlinear terms

56 $$\begin{aligned} \left\{ \begin{array}{l} \dot{X}(t)=A_0(\mu )X(t)+A_1(\mu )X(t-\tau )\\ \quad\quad\quad+\, G(X(t),X(t-\tau ),\mu ) \\ \dot{\mu }=0 \end{array}\right. \end{aligned}$$

where

$$D_{j+1}g(0,0,\mu _0) = [(D_{j+1}g)_{ik}]_{3\times 3}$$

and

$$A_j(\mu _0) = [(D_{j+1}g)_{ik}]_{2\times 2}$$

where $j=0,1$ and $i,k=1,\ldots ,3$. Here, $D_jg$ means the jacobian of *g* with respect to its *j*th component and $A_j(\mu _0)$’s are the submatrix of the matrix $D_{j+1}g(0,0,\mu _0)$.

Let ${C}=C([-\tau ,0],R^{2+1})$ be the Banach space of all continuous mappings from $[-\tau ,0]$ into $R^{2+1}$ which is equipped with the supremum norm $\Vert \phi \Vert _{\tau }=\sup _{\theta \in [-\tau ,0]}| \phi (\theta )|$ for $\phi \in {C}$.

We write the system (56) in the following DDE form

57 $$\frac{d}{dt}{U}(t)={L}_\mu U_t + F(U_t)$$

where  $$U_t(\theta )=[u(t+\theta ),\mu (t+\theta )]^T \in {C}$$ for  $$\theta \in [-\tau ,0]$$.

${L}{:}{C}\rightarrow R^{2+1}$ is the linear mapping and  $F \in C^r({C}, R^{2+1})$, $r\ge 1$ is the nonlinear mapping. Let $u(t)=X(t)$ and $u_t(\theta ) = u(t+\theta )$, then the system (56) is

58 $$\begin{aligned} \left\{ \begin{array}{l} \frac{d}{dt}u(t)=L_\mu u_t + G(u_t,\mu ) \\ \frac{d}{dt}\mu =0. \\ \end{array}\right. \end{aligned}$$

Therefore, for every $\varphi = (\varphi _1, \varphi _2)$ and $\phi = (\varphi ,\varphi _3)^T\in {C}$, we have

$$\begin{aligned}&A_0(\mu )\varphi (0)+A_1(\mu )\varphi (-\tau )=L_\mu \varphi \\&(L_\mu \varphi , 0)^T= {L}_\mu \phi \end{aligned}$$

and

$$F(\phi ,\mu )= (G(\varphi ,\mu ),0)^T.$$

The stability of the trivial solution of the Eq. (55) can be studied by the DDE of the following form

59 $$\begin{aligned} \left\{ \begin{array}{l} \dot{X}(t)=A_0(\mu )X(t)+A_1(\mu )X(t-\tau ) \\ \dot{\mu }=0. \\ \end{array}\right. \end{aligned}$$

Substituting $Y(t) = C e^{\lambda t}$ in the system (59), gives the following characteristic equation

60 $$\lambda \cdot \det (\lambda I_2-A_0(\mu )-e^{-\lambda \tau }A_1(\mu ))=0.$$

Obviously, the Eq. (60) always has one eigenvalue on the imaginary axis. We assume that this characteristic equation has $m+1$ eigenvalues (counting multiplicity) on the imaginary axis and all other eigenvalues have negative real parts. Therefore, the space *C* can be split as ${C} =P \oplus Q$ where $Q\subset {C}$ is infinite-dimensional stable subspace and $P\subset {C}$ is an (m + 1)-dimensional center subspace tangent to the center manifold. We will denote a basis for P by the $3 \times (m + 1)$ matrix $\varPhi$; the columns of $\varPhi$ are the basis vectors. Also, we will consider the transpose of the Eq. (59) with (m +1)-dimensional center subspace $P'$. We will denote a basis for $P'$ by the $(m + 1)\times 3$ matrix $\varPsi '$. Also, we define a new basis $\varPsi$ by $\varPsi =<\varPsi ',\varPhi >^{-1}\varPsi '$ which implies $<\varPsi ,\varPhi >= I$. This bilinear form is defined

61 $$\begin{aligned} <\psi _i,\phi _j> = \overline{\psi _i(0)}\phi _j(0)+ \int _{-\tau }^{0} \overline{\psi _i(\xi +\tau )}A_{1}\phi _j(\xi )d\xi \end{aligned}$$

where

$$\begin{aligned} \varPhi &= {} (\phi _1, \phi _2, \ldots , \phi _{m+1}) \\ \varPsi &= {} (\psi _1, \psi _2, \ldots , \psi _{m+1})^T \\ <\varPsi ,\varPhi > &= {} [<\psi _i, \phi _j>]_{(m+1)\times ( m+1)} \end{aligned}$$

This kind of basis $\varPsi$ can help us to decompose the space *C* and also reduce the Eq. (57) on the local center manifold $W^c_{loc}$ which is defined by

62 $$\begin{aligned} \begin{array}{l} W^c_{loc} = \{\phi \in {C}: \phi = \varPhi z + h(z,F),\\ \qquad \qquad z~ \hbox {is in a neighborhood of zero in } R^{m+1}\} \end{array} \end{aligned}$$

where $h(z,F) \in Q$ for each *z* and is a $C^{r-1}$ function with respect to *z*. Moreover, *z* satisfies the following ordinary differential equation

63 $$\frac{ d}{dt}z = Bz + \varPsi (0) F(\varPhi z +h(z,F))$$

where the $(m+1)\times (m+1)$ matrix *B* satisfies the relation $\frac{d}{d\theta }\varPhi =\varPhi B$, (Balachandran and Kalmar-Nagy 2009; Hale and Lunel 1993; Hale 1977).

## References

[CR1] Atangana A (2015). A novel model for the lassa hemorrhagic fever: deathly disease for pregnant women. Neural Comput Appl.

[CR2] Atangana A, Alkahtani BST (2015). Modeling the spread of Rubella disease using the concept of with local derivative with fractional parameter. Complexity.

[CR3] Atangana A, Goufo EFD (2014) On the mathematical analysis of Ebola hemorrhagic fever: deathly infection disease in West African countries. BioMed Res Int. 7 pages, Article ID 261383 10.1155/2014/261383 PMC4321856 25688348

[CR4] Balachandran B, Kalmar-Nagy T (2009). Delay differential equations, recent advances and new directions.

[CR5] Dadi Z, Afsharnezhad Z, Pariz N (2012). Stability and bifurcation analysis in the delay-coupled nonlinear oscillators. Nonlinear Dyn.

[CR6] Fenton A, Lello J, Bonsall MB (2006). Pahtogen responses to host immunity: the impact of time delays and memory on the evolution of virulence. Proc R Soc B Biol Sci.

[CR7] Guckenhiemer J, Holmes P (1993). Nonlinear oscillations, dynamical system, and bifurcations of vector fields.

[CR8] Hale J, Lunel S (1993). Introduction to functional differential equations.

[CR9] Hale J (1977). Theory of functional differential equations.

[CR10] Komarova NL, Baranes E, Klenerman P, Wodarz D (2003). Boosting immunity by antiviral drug therapy: a simple relationship among timing, efficacy, and success. Proc Natl Acad Sci USA.

[CR11] Li M, Shu H (2010). Global dynamics of a mathematical model for HTLV-I infection of $$ CD4^{+} $$ T cells with delayed CTL response. Nonlinear Anal Real World Appl.

[CR12] Perko L (1991). Differential equation and dynamical systems.

[CR13] Shu H, Wang L, Watmough J (2014). Sustaind and transient oscillation and chaos induced by delayed antiviral immune response in a immunosuppressive infection model. J Math Biol.

